# Mask-Wearing Perception of Preschool Children in Korea during the COVID-19 Pandemic: A Cross-Sectional Study

**DOI:** 10.3390/ijerph191811443

**Published:** 2022-09-11

**Authors:** Miji Kwon, Eun-Mi Jang, Wonyoung Yang

**Affiliations:** 1 Department of Speech-Language Rehabilitation & Counseling, Gwangju University, Gwangju 61743, Korea; 2 Department of Clinical Psychology, Daegu Cyber University, Gyeongsan 38453, Korea; 3 Division of Architecture, Gwangju University, Gwangju 61743, Korea

**Keywords:** COVID-19, face masks, preschool children, mask-wearing perception, physical self-perception, Korea

## Abstract

As the COVID-19 pandemic continues, wearing a mask has become a daily routine in Korea over the last two years. This study aims to investigate the mask-wearing perception of preschoolers (ages 4–6). The questionnaire comprised 17 yes-no closed-ended questions and two open-ended questions, and interviews of the children were conducted from January to February 2022, 15 months after mandatory mask wearing. Results showed that children were aware of the need to wear a mask to protect themselves and others from the coronavirus, and they perceived it as necessary and a good thing. Most children responded that they did not feel uncomfortable wearing a mask at preschool. This perception was thought to be influenced by the caregivers’ perceptions of the mask in Korea. The way in which 4–5-year-olds perceived the mask differed from the way 6-year-olds did. Children aged between four and five seemed to perceive the mask as a physical self, while children aged six did not. As children who have experienced COVID-19 are growing up, attention is being focused on how the experience of wearing a mask affects their early childhood development.

## 1. Introduction

Since the World Health Organization (WHO) declared an international public health emergency on 30 January 2020, and a pandemic on 11 March 2020 [1], wearing a mask in public places has become the new normal in Korea to reduce the spread of the virus. Domestic mask production and purchase were unstable in the early days of the pandemic. After a while, both stabilized enough in Korea to recommend wearing a mask in public transportation and places on 25 May 2020 [2]. As of 13 November 2020, wearing face masks had become compulsory in public places [3]. After ensuring the outbreak was sufficiently controlled, finally, wearing a mask outdoors has been made voluntary rather than mandatory since 2 May 2022 [4].

Mask-wearing behavior varies according to cultural [5,6,7], political [8,9], and health-related [10,11] concerns. According to the snap poll conducted in 28 countries by Gallup International [12], the results of which were reported on 26 March 2020, 94% of Koreans stated that they used medical masks as a precaution adopted to protect themselves from SARS-CoV-2. Among the 28 countries, the percentage of people who wore a face mask was the highest in Korea (94%), followed by Thailand (81%) and Japan (70%). On the other hand, 1% of Dutch people and 3% of Germans responded that they wore face masks for a precautionary measure. East Asian countries are more open to wearing face masks than European or American countries [13].

The mask-wearing behaviors of Koreans have been intensively investigated during the COVID-19 pandemic. Ha [14] proposed that there were three phases of change in awareness regarding wearing a face mask in 2021, namely, familiarly wearing, frequently wearing, and always wearing. In Korea, mask wearing was considered a seasonal behavior due to the yellow dust originating from the deserts in Central Asia during the spring season. During the Middle East respiratory syndrome coronavirus (MERS-CoV) outbreak in 2015, there was an increase in public awareness regarding the use of face masks. During the COVID-19 pandemic, however, Koreans have had to always wear face masks instead of only when they felt the need to do so. Kang et al. [15] observed the type of masks worn and the proper wearing of masks in Seoul’s cafes, supermarkets, underground shopping malls, and streets in August (social distancing levels 1 and 2), October (social distancing level 1), and November 2020 (after the mask mandates were passed). In August 2020, 22.1% of subjects in cafes, 90.8% in supermarkets, 91.8% in underground shopping malls, and 83.6% on outdoor streets wore masks properly. In October 2020, the proportion of people wearing masks correctly increased at all locations. After the mask mandate was implemented in November 2020, about 97% of subjects of supermarkets, underground shopping malls, and streets wore masks properly. In cafes, the proportion of people wearing masks correctly was 61.5% under both social distancing level 2 in August and the mandate in November. Chang et al. [16] conducted a cross-cultural comparison study between the United States (*n* = 150) and Korea (*n* = 150). They found that Americans needed to be encouraged to wear masks voluntarily while Koreans perceived the social benefits of mask wearing. Chung et al. [17] investigated the mask-wearing behavior of Koreans based on the theories of individualism–collectivism. Two online surveys were conducted on 9 July 2020 (*n* = 1000) and 21–29 December 2020 (*n* = 1569). They concluded that the general collectivism of Korean people was mutually synergistic with their horizontal individualism in responding to the COVID-19 pandemic in Korea. Mo and Park [18] found that horizontal individualism, vertical collectivism, and cognitive and affective we-ness positively influenced mask-wearing behavior in their survey study (*n* = 720). Kim and Han [19] found, using an online survey with 280 adults, that mask use could be expected to increase further if people perceived a personal need to wear masks, if their peers perceived the importance of mask use, and if they possessed civic consciousness that considered society as a whole.

Always mask-wearing in Korea has been considered a successful control measure for COVID-19 over the last two years without a strong lockdown policy [20]. However, in the short term, complications with face masks lead to increased risk of aspiration, difficulty in receiving the required fraction of inspired oxygen, painful facial trauma, difficulty in the expectoration of secretions, difficulty in communicating, claustrophobia, etc. [21]. Nationwide mask wearing over two years could adversely affect young children in the long term. However, studies on the negative effects of long-term mask wearing on young children are still in their early stages. In their preprint of a longitudinal study of children born during the pandemic period in the U.S., Deoni et al. [22] provided preliminary evidence of reduced verbal performance among them compared with pre-pandemic born children, even though mask wearing was not the only factor in the study. Face masks disrupt holistic processing and face perception in children aged 6–14 [23]. Duran [24] investigated Turkish preschool children’s perception of the COVID-19 pandemic through their drawings and individual interviews. The children knew what the 2019 Coronavirus was, how it infected people, and how they could protect themselves from the virus with measures such as wearing masks or washing their hands with soap. The quarantine-forced school closure significantly affected primary school children’s physical activity, quality of sleep, psychological status, eating habits, academic performance, and household income in Greece [25]. In Korea, where always mask-wearing became the new normal, the mask-wearing behavior of young children in their early development has not yet been widely investigated. Kim [26] examined the phenomenological meaning of face mask wearing under the prolonged COVID-19 pandemic with nine children aged four in a preschool from April to July 2021. She derived five themes using a hermeneutic phenomenological approach, namely, a mask that my body became familiar with but still [finds] strange, a means of expressing oneself, awareness of relationship through masks, and mask-wearing followed by internalized fear. It is widely known that changes in language development are rapid up to the age of 3.5 years [27]. The potential impact of mask-wearing on early-childhood language and speech development could be critical. It has been reported that children aged 6 to 7 also tend to have lower word recognition skills due to wearing a mask [28]. On the other hand, there are no known studies that use of a face mask negatively impacts a child’s speech and language development [29]. Conflicting views have been reported on the effect of mask wearing on children’s language development.

The potential impact of mask wearing specifically on early childhood development will be observed from now onward. As the first step, it is essential to investigate the mask-wearing behavior of young children at various points in time during the COVID-19 pandemic. Furthermore, the mask-wearing behavior of adults at the same points in time in Korea should be investigated simultaneously. This is because there is well-documented and consistent evidence that the child and the culture are mutually interactive systems [30].

The purpose of this study was to capture the mask-wearing perceptions of preschool children in Korea after a mask-wearing period spanning two years. It can be used as a basis for research on mask-wearing behavior of preschool children and on the various effects of long-term mask-wearing on children’s development.

## 2. Materials and Methods

### 2.1. Participants

Seventy-four preschoolers (ages 4–6) were recruited with parental consent, mostly from two preschools in Gwangju, Korea. The children received toys and books as compensation for their participation. The institutional review board of Gwangju University approved the informed consent procedure. Table 1 lists the number of participants by age.

### 2.2. Research Methodology and Questionnaire Design

The questionnaire comprised three sections, namely, COVID-19 awareness, daily practice, and face mask perception. The section on face mask perception was developed with four items, namely, necessity, wearing, discomfort, and likeability, based on Kim’s phenomenology research [26] on children’s mask-wearing behaviors. A panel of three experts (professor of early childhood education, early childhood teacher, and psychotherapist) evaluated the draft questionnaire, whether the questions according to the purpose of the study were suitable for the criteria of children’s developmental psychology and children’s language development. Following the panel review, the questionnaire items were modified using colloquial language that is easy for four-year old children who were the youngest in this study to understand. Table 2 lists the contents of questionnaire questions and types. Seventeen were yes–no questions, as listed in Table 3. Two were open-ended questions to ask necessity of the mask-wearing and discomfort due to the mask-wearing. Finally, prior to the on-site interview, preliminary interviews were conducted with two children aged 4, 5, and 6 each, and at this time, the final revision was made in colloquial language that children could understand.

A methodological triangulation [31], which is a method of obtaining complementary findings [32], was applied for the questionnaire design. Closed-ended questions, which were used most frequently in developmental studies involving preschool children [33], were mainly used to achieve quantitative data from children, and open-ended questions were complemented to evaluate children’s mask-wearing perception qualitatively and to understand potential children’s response tendencies to yes–no questions [34,35,36].

### 2.3. Interviewer Training and Interview Procedure

A training session was held for the interviewers a week before the interviews commenced. Three authors (two university professors and a former preschool teacher), functioning as interviewers, shared a brief description of the study, structure of the survey questionnaire, directions on how to build rapport, and instructions on procedure.

The one-to-one interviews were conducted between 25 January and 28 February 2022 in a classroom assigned to a preschool during school activities. At the time of the interviews, almost 23 months had passed since mask wearing commenced in Korea because of the COVID-19 pandemic, and it had been 15 months since the mask mandate in public spaces, including preschools, was implemented. The preschool’s COVID-19 quarantine measures were observed (thermal check/hand sanitization/mask-wearing at all times), and temperature checking, sanitizing hands, and wearing a mask at all times before the experiment were conducted. Both the child and the interviewer wore face masks during the interview. The interviewer asked questions or commented on the child’s personal life, school, and hobbies for rapport-building [37]. The interviewer gave the “I don’t know” instruction before starting the questionnaire to reduce children’s suggestibility to misleading questions [38]. The interviewer then took the time to personally ask each child all the questions in the questionnaire. The interview duration was based on attention in early development [39,40,41]. Although the interview time differed depending on the child, it was not allowed to exceed a maximum of 7~10 min.

The interviewers asked the preschoolers 19 questions based on the questionnaire prepared. Seventeen were yes–no questions, as listed in Table 3. Two were open-ended questions: “Why do you wear a mask?” and “What are the discomforts of wearing a mask?” All interviews were audio recoded.

### 2.4. Data Analysis

For quantitative data, the frequency and percentage were calculated using descriptive statistics through the SPSS Statistics 27, and the chi-test was performed to examine the differences according to age.

For qualitative data analysis, content analysis was conducted based on interview data with children. For data analysis, we started by reading all the data over and over again, concentrating on the interview data with children. Then, words containing core ideas and concepts were listed, categorized, and reclassified. 

## 3. Results

### 3.1. Closed-Ended Questions

The children’s yes-no responses were analyzed by age and gender. Only age showed a statistical significance. Table 3 lists the responses of the four- to six-year-olds. Of them, 89.2% answered that they knew what the 2019 Coronavirus was. The majority of them (98.6%) wanted “the Coronavirus to go away.” Three quarters of them thought the pandemic would get worse, but only 56.8% said that they wanted to stay home because they were afraid of the virus. Most of them had daily practices for preventing the spread of COVID-19. They even tried to avoid touching the mask when they were wearing it.

In total, 87.8% of the children responded that they needed a mask, and 83.8% said that a mask was to be worn every day. All the children excluding one answered that masks must not be taken off at preschool, although 47.3% wanted to take off the mask. Only one-third of the children said that masks were uncomfortable, but two-thirds felt uncomfortable when conversing with their friends in a low voice. A greater number of children said that wearing a mask was cool than those who said that it is cool when they take off the mask. In addition, 90.5% of the children said that a mask was a good thing. 

The responses for the four questions, excluding the “I don’t know” one, showed a statistically significant age effect, as listed in Table 4. The responses of the 4- to 5-year-olds for the four questions differed from those of the 6-year-olds. The number of 4-year-olds and 5-year-olds who said they wanted to take off the mask was lower than the number of children who said they did not want to take off the mask. On the other hand, two-thirds of the 6-year-olds wanted to take off the mask. A majority of the 4- to 5-year-olds answered that wearing a mask all day was not uncomfortable and that they even looked cool with a mask on. However, the 6-year-olds showed no preference. When asked whether the mask is a good thing, 100% of the 4- to 5-year-olds said yes, but not all the 6-year-olds answered yes.

### 3.2. Open-Ended Questions 

Table 5 and Table 6 organize the children’s responses to the open-ended questions. The children responded with phrases, complete sentences, or sometimes nodding or shaking their heads.

The five categories derived through content analysis from ‘Why do you wear a mask’ are as follows: “coronavirus”, “infection”, “symptom”, “pathogenesis”, and “anticipation”. The children were aware of the need to wear a mask because of the COVID-19 pandemic. As the age of the respondents increased (from 4 to 5 to 6 years old), they responded more specifically about the reason. For example, we talked about specific topics regarding droplet transmission and aetiology, such as the Coronavirus must not enter our body, and the disease could be caught if the virus entered the mouth and nose. In addition, we also talked about anxiety about fine dust, yellow dust, and mutated viruses.

The five categories derived through content analysis from ‘Is there any discomfort when wearing a mask?’ are as follows: “no discomfort”, “little discomfort”, “ear pain”, “difficulty in breathing”, “difficulty in hearing voices”. Most of the 4- and 5-year-olds answered “no” regarding the discomfort of the mask, but the 6-year-olds specifically reported difficulties in wearing the mask, such as “stuffiness, nose pain, ear pain, and difficulty hearing.” In addition, 82.4% of the children said that they felt no discomfort when wearing a mask with their subjective response. Further, 67.6% of the children replied that masks are not uncomfortable and difficult to wear in the yes–no question. The discrepancy between the yes–no and open-ended question might arise depending on the type of questions, but most children responded that they did not feel uncomfortable wearing a mask.

## 4. Discussion

### 4.1. Effects of Caregivers

The children answered that the pandemic would worsen and they wanted to stay home because of it. However, in general, they showed no negative feelings toward a face mask. Even though half of them wanted to take off the mask, they said that they were not uncomfortable wearing a mask and that they even looked cool with a mask on. The fear about the pandemic did not directly affect the children’s perception of masks negatively. This could be due to the behaviors of the caregivers. Children are marginalized in adult-centered society [42]. 

The age of the subjects of this study was between 4 and 6 years old, and their age at the time of the COVID-19 outbreak was approximately between 2 and 4 years old. Parents who form attachments to children aged between 2 and 4 are important factors in social adaptation [43]. Bowlby defined attachment as lasting psychological connectedness between human beings [44], and caregivers provide safety and security for the infant [45]. Cho et al. [46] found that infants, toddlers, and young children understood the COVID-19 situation through their parents’ reactions. According to their report, even young children who did not know much about COVID-19 could understand infectious diseases by seeing the reactions of their parents, to whom they were attached. It can be understood that the sensitive or insensitive response of parents to infectious diseases especially affects young children. Lim et al. [47] described the experience of managing infectious diseases through voluntary public participation in Korea. Wearing a mask was understood as a fundamental way to avoid doing harm to others. Before the mask mandate in public places was implemented (13 November 2020), more than 90% of the people in supermarkets (96.0%), underground shopping malls (94.7%), and even outdoor streets (91.1%) wore masks properly in Seoul, according to the 15 October 2022 data from the Kang et al. [15] study. No intense protests or lawsuits regarding the wearing of masks occurred in Korea, as they did in some countries such as the U.S. and some European countries [13,47]. The children in this study may have been affected by the perception of their parents or teachers about COVID-19, who socially accepted wearing masks.

As COVID-19 continues for a long time, caregivers should follow the government’s policy, but show a consistent and flexible attitude without reacting sensitively.

### 4.2. Likeability of Face Mask

A total of 67.6% of the 4- to 6-year-olds in this study thought they looked cool with a mask on and 90.5% of the children answered that the mask was a good thing. This is consistent with the recently published phenomenological study by Kim [26] in Korea, which was the only reference regarding preschool children’s perception of the mask during the COVID-19 period, to the best of our knowledge. Kim observed nine 4-year-olds in the same preschool class from April to July 2021 wearing masks for more than a year. These children assumed it was normal to wear a mask in preschool. Initially, they found it uncomfortable to wear a mask, but later, breathing and talking were not uncomfortable. Children often forgot to remove their masks while brushing their teeth or eating snacks or lunch. They even expressed preferences regarding the mask, such as with color or pattern, and found commonalities in each other’s masks. Face masks were considered by children to be a part of their body (but still strange) and were considered a means of expressing themselves and building relationships.

Our results show that 4- and 5-year-olds seemed to have a strong physical self-perception about wearing a mask, but 6-year-olds do not. It is reminiscent of the physical self-concept as a body substance [48]. Physical self-perceptions may help advance research on the established hierarchical links with higher order constructs such as self-esteem and other behaviors [49,50]. The physical self-concept develops before other self-concepts, and positive thoughts about one’s body can affect the development of self-concepts in other areas [51]. Since children between the ages of 4 and 5 have been wearing masks on a daily basis since around the age of 2 or 3, when their physical self-concept develops, they become rather anxious if they do not wear a mask.

### 4.3. Difference by Age: Physical Self-Perception

The difference in responses between the 4–5-year-olds and the 6-year-olds seemed to be due to the difference in the cognitive development process of children of that age. According to Piaget’s theory of cognitive development [52], children of this age are classified as preoperational. Preoperational children are capable of thinking, but at an intuitive level, are not yet logical, and have egocentric thinking, believing that other people’s thoughts and feelings are the same as their own. Therefore, it was found that there is no negative perception of mask wearing owing to the parents’ attitude toward COVID-19 and masks and the education provided by the preschool. The 6-year-olds in this study appeared to have entered the concrete operational stage. Unlike the 4–5-year-olds, they did not show an unconditional positive reaction to the mask. By acquiring a basic logical system, children in the concrete operational stage can think theoretically and logically, centered on concrete objects, and can be decentralized by recognizing that other people’s viewpoints and thoughts may differ from their own.

Moreover, Piaget divided the stages of moral development into heteronomous and autonomous morality [53]. Children in the heteronomous morality stage, which corresponds to the preoperational moral level, unilaterally obey the rules set by adults, and the rules are absolute and unchangeable. Autonomous morality corresponds to the concrete operational stage, and children recognize the fact that rules are made by mutual agreement and can be changed, and make judgments based on whether their intentions are right or wrong rather than the result of their actions itself. This stage of early childhood morality also explains the results of the children’s perception of masks by age.

### 4.4. Limitations and Future Studies

The present study was conducted in two randomly chosen preschools. Socioeconomic status and parenting were not considered in the choice of participants. The fact that mask-wearing continued for two years, including the government’s 15-month mandatory wearing period, could undermine the effect of socioeconomic status on mask-wearing behavior of young children.

The number of children in each age group may be insufficient to generalize the results. Considering the situation at the time of the interviews, access to children during the COVID-19 pandemic was the biggest obstacle to the study.

The potential impact of long-term mask wearing on children is currently beginning to be studied. In particular, the effects of the mask on speech and language development of children who have been wearing masks every day for more than 2 years during their language development stage should be closely investigated from now on. Recently, taking off a mask has become an awkward atmosphere for teenagers, and a neologism called ‘Ma-gi-gun’ (mask + scammer, a neologism meaning that the difference between wearing and taking off a mask is the level of fraud) [54] has appeared in Korea. This neologism reflects that the mask-wearing perception is not only a matter of early childhood but also a problem of adolescence. Furthermore, it shows that the mask as a physical self-perception could be extended to the perception of others. 

It is predicted that Korea and the world will continue to wear masks, although there may be differences in place and time of use for the purposes of new infectious diseases, fine dust, and yellow dust. It is necessary to study how the perception of masks will change as children who have experienced the COVID-19 pandemic grow up.

## 5. Conclusions

As the COVID-19 pandemic continues, wearing a mask has become a daily routine in Korea over the last two years. Face masks are compulsory even in preschools during the COVID-19 pandemic. In this study, the perception of wearing a mask for preschoolers aged 4-6 years was investigated at the time of mandatory mask wearing.

Preschoolers were aware of the need to wear a mask to protect themselves and others from the coronavirus, and they perceived it as necessary and a good thing. Most children responded that they did not feel uncomfortable wearing a mask at preschool all day long. This perception was thought to be influenced by the caregivers’ perceptions of the mask in Korea. 

The way in which 4–5-year-olds perceived the mask differed from the way 6-year-olds did. The 4–5-year-olds perceived the mask as a physical self, while children aged six did not. Most children aged four and five responded they looked cool with a mask on, did not want to take off, and did not feel uncomfortable. All children aged 4–5 said that a mask was a good thing, but not all 6-year-olds said it was good. 

As children who have experienced COVID-19 are growing up, attention is being focused on how the experience of wearing a mask affects their early childhood development.

## Figures and Tables

**Table 1:** Description of the participants.

Age	4 Years		5 Years		6 Years		Sub Total		Total
Gender	Girl	Boy	Girl	Boy	Girl	Boy	Girl	Boy	
Number	9	8	18	12	14	13	41	33	
(%)	(53%)	(47%)	(60%)	(40%)	(52%)	(48%)	(55.4%)	(44.6%)	
Sub Total	17		30		27				74
(%)	(23%)		(40.5%)		(36.5%)				(100%)

**Table 2:** Contents of questionnaire questions and types.

Category	Number of Questions	Type of Question
COVID-19 Awareness	5	Closed-ended question (Yes–No)
Daily Practices for Preventing the Spread of COVID-19	3	Closed-ended question (Yes–No)
Mask Perception	9	Closed-ended question (Yes–No)
Necessity of mask-wearing	1	Open-ended question
Discomfort due to mask-wearing	1	Open-ended question
Total	19	

**Table 3:** Questionnaire and the children’s responses (*: a significant age difference).

Category		Questionnaire	Yes		No		I Don’t Know.	
			*n*	(%)	*n*	(%)	*n*	(%)
COVID-19 Awareness		I know what Coronavirus is.	66	(89.2)	8	(10.8)		
		I think Coronavirus will get worse.	56	(75.7)	17	(23.0)	1	(1.4)
		I want Coronavirus to go away.	73	(98.6)	1	(1.4)		
		I want to stay at home because I am afraid of Coronavirus.	42	(56.8)	30	(40.5)	2	(2.7)
Daily Practices for Preventing the Spread of COVID-19		I wash my hands often.	70	(94.6)	4	(5.4)		
		I wear the mask correctly.	72	(97.3)	2	(2.7)		
		I do not touch the mask often.	17	(23.0)	57	(77.0)		
Mask Perception	Necessity	I need a mask.	65	(87.8)	7	(9.5)	2	(2.7)
	Wearing	A mask is to be worn every day.	62	(83.8)	9	(12.2)	3	(4.1)
		Masks must not be taken off at daycare.	1	(1.4)	73	(98.6)		
		I want to take off the mask. *	35	(47.3)	39	(52.7)		
	Discomfort	Masks are uncomfortable and difficult to wear	24	(32.4)	50	(67.6)		
		Wearing a mask all day is not uncomfortable. *	22	(29.7)	52	(70.3)		
		Masks are uncomfortable when talking in a low voice with friends.	28	(37.8)	45	(60.8)	1	(1.4)
	Likeability	I look cool with a mask off.	39	(52.7)	31	(41.9)	4	(5.4)
		I look cool with a mask on. *	50	(67.6)	20	(27.0)	4	(5.4)
		A mask is a good thing. *	67	(90.5)	7	(9.5)		

**Table 4:** Children’s results with a significant age difference (*: *p* < 0.05, **: *p* < 0.01).

Mask Perception		Reponses	4-Year-Olds	5-Year-Olds	6-Year-Olds	χ² (*p*)
			*n* (%)	*n* (%)	*n* (%)	
Wearing	I want to take off the mask.	Yes	7 (41.2)	10 (33.3)	18 (66.7)	6.666 (0.036) *
		No	10 (58.8)	20 (66.7)	9 (33.3)	
		Sub Total	17 (100)	30 (100)	27 (100)	
Discomfort	Wearing a mask all day is not uncomfortable.	Yes	4 (23.5)	4 (13.3)	14 (51.9)	10.498 (0.005) **
		No	13 (76.5)	26 (86.7)	13 (48.1)	
		Sub Total	17 (100)	30 (100)	27 (100)	
Likeability	I look cool with a mask on.	Yes	12 (75.0)	25 (89.3)	13 (50.0)	10.325 (0.006) **
		No	4 (25.0)	3 (10.7)	13 (50.0)	
		Sub Total	16 (100)	28 (100)	26 (100)	
	A mask is a good thing.	Yes	17 (100)	30 (100)	20 (74.1)	13.458 (0.001) **
		No	0 (0)	0 (0)	7 (25.9)	
		Sub Total	17 (100)	30 (100)	27 (100)	

**Table 5:** Children’s responses to subjective question No. 1: Why do you wear a mask?

4-Year-Olds (17)		5-Year-Olds (30)		6-Year-Olds (27)		Total Count	Keyword Derived
Children’s Responses	Count	Children’s Responses	Count	Children’s Responses	Count		
“Because of COVID-19 (Coronavirus).”	6 (35.3%)	“Because of COVID-19 (Coronavirus).”	17 (56.7%)	“Because of COVID-19 (Coronavirus).”	7 (25.9%)	30 (40.5%)	“Coronavirus”
“Because of the chances of being infected by COVID-19.”	4 (23.6%)	“Because of the chances of being infected by COVID-19.”	4 (13.3%)	“Because of the chances of being infected by COVID-19.”	6 (22.2%)	14 (18.9%)	“Infection”
“Because of getting sick because of COVID-19 infection.”	1 (5.9%)	“Because I have to stay in a hospital without seeing Mom.”	1 (3.3%)	“When I get COVID-19, I have a fever and hate it.” “If the Coronavirus enters my mouth or nose, I can get sick.”	2 (7.4%)	4 (5.4%)	“Symptom”
“Because cold germs come in.” “Because Coronavirus enters my body, so it needs to be worn.” “When you touch people, you can get COVID-19.”	3 (17.6%)	“It should not enter our bodies.” “It enters our noses and mouths.” “Because it gets in my mouth and causes a headache, fever, and cough.”	4 (13.3%)	“If virus and fine dust become severe, a mutated virus may appear.” “If you do not wear a mask, you can get a virus.” “You should not spit. You can spread COVID-19.” “Coronavirus, fine dust, and Omicron virus must not enter the body.” “Prevention of COVID-19.” “I am afraid I will catch the Coronavirus if it gets in my mouth.” “I am afraid of the Coronavirus entering my mouth and nose.” “When you touch people, you can get COVID-19.”	9 (33.3%)	14 (18.9%)	“Pathogenesis”
“I hope COVID-19 goes away.”	1 (5.9%)	“I hope COVID-19 goes away forever.” “COVID-19 can get worse.”	3 (10.0%)	“COVID-19 can get worse.”	1 (3.7%)	5 (6.8%)	“Anticipation”
“I like masks.” “You must wear a mask when you go out.” “I do not use it at home.”	3 (17.6%)	“There are bacteria and dust.”	1 (3.3%)		0	4 (5.4%)	Miscellaneous
	0		0	“I do not know.”	1 (3.7%)	1 (1.4%)	
Non-response	0	Non-response	0	Non-response	1 (3.7%)	1 (1.4%)	Non-response

**Table 6:** Children’s responses to subjective question No. 2: What are the discomforts of wearing a mask?

4-Year-Olds (17)		5-Year-Olds (30)		6-Year-Olds (27)		Total Count	Keyword Derived
Children’s Responses	Count	Children’s Responses	Count	Children’s Responses	Count		
“I have no discomfort.” “It’s not uncomfortable.”	16 (94.1%)	“I have no discomfort.” “It’s not uncomfortable.”	26 (86.7%)	“I have no discomfort.” “It’s not uncomfortable.”	19 (70.4%)	61 (82.4%)	“No discomfort”
“It’s a little uncomfortable.”	1 (5.9%)	“Uncomfortable when wearing it.”	1 (3.3%)	Non-response	0	2 (2.7%)	“A little discomfort”
Non-response	0	Non-response	0	“My ear hurts.” “My ears feel uncomfortable and it hurts a little bit.”	3 (11.1%)	3 (4.1%)	“Ear pain”
Non-response	0	“Feeling stifled and it’s uncomfortable on my nose.”	1 (3.3%)	“Feeling stifled.” “Hard to breath.”	2 (7.4%)	3 (4.1%)	“Difficulty in breathing”
Non-response	0	Non-response	0	“Hard to hear voices.”	2 (7.4%)	2 (2.7%)	“Difficulty in hearing voices”
Non-response	0	“I cannot scratch my chin when itchy because COVID-19 does not go away.”	2 (6.7%)	“It keeps going down.”	1 (3.7%)	3 (4.1%)	Miscellaneous

## Data Availability

Not applicable.
